# Neuroregeneration in neurodegenerative disorders

**DOI:** 10.1186/1471-2377-11-75

**Published:** 2011-06-23

**Authors:** Ana M Enciu, Mihnea I Nicolescu, Catalin G Manole, Dafin F Mureşanu, Laurenţiu M Popescu, Bogdan O Popescu

**Affiliations:** 1 Department of Cellular and Molecular Medicine, 'Carol Davila' University of Medicine and Pharmacy, School of Medicine, 8 Eroilor Sanitari, sector 5, Bucharest 050474, Romania; 2 Laboratory of Molecular Medicine, 'Victor Babeş' National Institute of Pathology, 99-101 Splaiul Independenţei, sector 5, Bucharest 050096, Romania; 3 Department of Neurology, 'Iuliu Hatieganu' University of Medicine and Pharmacy, 8, Victor Babeş, Cluj Napoca 400023, Romania; 4 Department of Neurology, University Hospital Bucharest, 'Carol Davila' University of Medicine and Pharmacy, 169 Splaiul Independenţei, sector 5, Bucharest 050098, Romania

## Abstract

**Background:**

Neuroregeneration is a relatively recent concept that includes neurogenesis, neuroplasticity, and neurorestoration - implantation of viable cells as a therapeutical approach.

**Discussion:**

Neurogenesis and neuroplasticity are impaired in brains of patients suffering from Alzheimer's Disease or Parkinson's Disease and correlate with low endogenous protection, as a result of a diminished growth factors expression. However, we hypothesize that the brain possesses, at least in early and medium stages of disease, a "neuroregenerative reserve", that could be exploited by growth factors or stem cells-neurorestoration therapies.

**Summary:**

In this paper we review the current data regarding all three aspects of neuroregeneration in Alzheimer's Disease and Parkinson's Disease.

## Background

**Figure 1:**
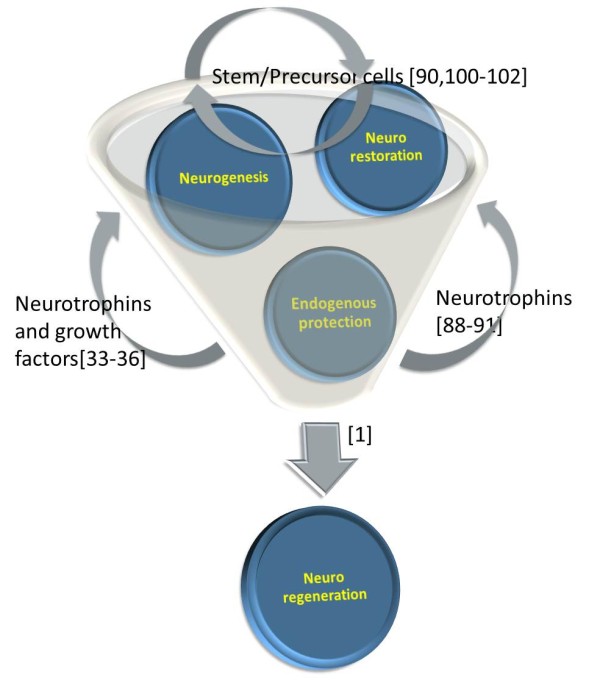
**The large concept of neuroregeneration contains three landmarks: endogenous protection by growth factors, neurogenesis and neurorestoration**. Rather than perceiving them as isolated events, they should be viewed interrelated, one creating the premises for generating the other.

Adult neuroregeneration is a complex concept, beyond the common knowledge of neurogenesis that also comprises endogenous neuroprotection leading to neuroplasticity and neurorestoration -a therapeutical approach of implantation of viable cells (Figure 1). Regeneration in the central nervous system (CNS) implies that new neurons, generated either through proliferation of endogenous stem/progenitor cells or by administration of exogenous stem/precursor cells with potential to substitute for lost tissue, will differentiate, survive, and integrate into existing neural networks [1]. Among the three components of neuroregeneration previously mentioned, neuroplasticity was the first one put forward, by Ramon y Cajal, in 1894: "associations already established among certain groups of cells would be notably reinforced by means of the multiplication of the small terminal branches of the dendritic appendages and axonal collaterals; but, in addition, completely new intercellular connections could be established thanks to the new formation of [axonal] collaterals and dendrites." [2]. However, Ramon y Cajal discards, in the same paper, the possibility of cell renewal: "it is known that the nerve cells after the embryonic period have lost the property of proliferation". Adult neurogenesis was proposed by Joseph Altman in the 1960's, in a series of articles involving tritiated thymidine retaining cells in the rat brain [3-5]. The newly emerged concept was a controversy until the early 1990s, when several reports [6-9] proved beyond doubt the existence of adult neural stem cells.

The concepts of neuroplasticity and neural stem cells led to the idea of neurorestoration as an alternative therapy for neurodegenerative disorders such as Alzheimer's Disease (AD) and Parkinson's Disease (PD), both characterized by neuronal loss. Our review will attempt to answer the question "Is there any neuroregeneration in neurodegeneration?" taking into account the three concepts mentioned above.

## Discussion

### Neurogenesis in neurodegenerative diseases

The adult mammalian brain retains a limited capacity of neurogenesis, which manifests in the subventricular zone (SVZ) and subgranular zone of the hippocampal dentate gyrus. The neuronal precursors migrate into the olfactory bulb, the granular cell layer, or, if necessary, to the striatum, CA1 region of hippocampus or cerebral cortex [10].

#### Alzheimer's Disease animal models

Neurogenesis in AD transgenic mice is usually impaired, but the results may differ from one transgenic strain to another [11]. Haughey et al. reported that proliferation and survival of neural precursor cells (NPC) was reduced in the dentate gyrus of APP mutant mice with already constituted amyloid deposits [12]. Furthermore, the decrement in NPC number was correlated with accumulation of Aβ, even in oligomeric, diffusible form [11]. Although Kolecki et al. confirmed the previous results, they reported that overexpressing APP and Aβ in transgenic mice do not interfere with the mitotic activity of NPC, as assessed by Ki-67 [13].

*In vitro*, Aβ effects reported on mouse brain-derived neurospheres are different with the type of peptide used: i) Aβ 25-35 induces neuronal differentiation and apoptosis in neural committed cells [14]; ii) Aβ40 promotes neurogenesis in NPCs [15]; iii) Aβ42 stimulates neurosphere formation and increases the number of neuronal precursors [16]; it also has a reported effect of inducing astrocytic differentiation [15].

#### Evidence of neurogenesis in AD human brain

An overexpression of neurogenesis markers (Doublecortin - DCX, Polysialylated Neural Cell Adhesion Molecule - PSA-NCAM and TUC-4) in hippocampus of AD patients, without a correlated increase in mature neuronal markers (NeuN, Calbinding D28k) is reported by Jin et al. [17]. This expression disjunction sustains the hypothesis of AD as a failed attempt of precursor cells to neuronal differentiation [18], but Boekhoorn et al argue that DCX is a nonspecific marker, increased due to reactive gliosis [19]. Furthermore, Verwer et al. questioned whether DCX+ cells are indeed neuroblasts, presenting arguments for their astrocytic origin [20]. Investigating Musashi1 immunoreactivity in SVZ of AD patients, Ziabreva et al. also reported impaired neurogenesis, as compared to controls [21]. In turn, although Lovell et al. isolated viable NSC from AD patients' hippocampi, they obtained decreased viable NPC yields and altered division rates, as compared to controls [22].

*In vitro *studies using human neurospheres reported, unlike *in vitro *models using rodent NPCs, that Aβ 1-40 treatment impaired proliferation and differentiation of precursor cells [23].

In order to assess neurogenesis in AD brain, adding to contradictory results in literature, one must further take into account the neurogenesis-stimulating effect of AD medication [24].

#### Neurogenesis in PD animal models

Adult mice *substantia nigra *contains bromodeoxyuridine (BrdU) incorporating cells that show dividing and differentiating properties. *In vivo*, this potential seems to materialize into glial lineage, whereas *in vitro*, under appropriate growth factors stimulation, neuronal progenitors may be identified [25]. Reports regarding neurogenesis in 6-hydroxydopamine (6-OHDA) models of PD showed increased number of BrdU+ cells and a tendency to migrate towards the lesioned striatal nuclei [26], but without further differentiation on neural lineage [27].

Transgenic mice overexpressing human mutated α synuclein exhibited reduced BrdU+ cells and decreased survival of newly generated neurons, as compared to aged-matched controls. Interestingly, the cessation of α synuclein overexpression led to recovered neurogenesis [28].

#### Neurogenesis in PD human brain

The numbers of proliferating cells in the subependymal zone and neural precursor cells in the subgranular zone and olfactory bulb are reduced in postmortem brains of Parkinson's Disease patients [29]. However, there are reports of newly generated neuroblasts PSA-NCAM + in *substantia nigra *of PD patients, without a solid proof of further dopaminergic neuronal differentiation or reintegration in neuronal circuitry [30].

### Endogenous neuroprotection and growth factors

Discovery of growth factors and their pro-survival effect led to a closer investigation of specific nervous system cytokines - Nerve Growth Factor (NGF), Brain-Derived Nerve Factor (BDNF), Glial-Derived Nerve Factor (GDNF) - involvement in the outcome of neurodegenerative diseases. Interestingly, different neuronal subpopulations require different growth factors to thrive, for example NGF protects cholinergic neurons from various insults [31], whereas for dopaminergic neurons, this effect is better sustained by BDNF [32].

Neurotrophins (NGF, BDNF, neurotrophin 3 - NT3 and neurotrophin 4 - NT 4) are most studied for their involvement in normal central nervous system (CNS) development [33-36] and in normal [37] or pathological ageing [38-40]. They exert their effect through tropomyosin-related kinase (Trk) receptors and activation of several signaling cascades: i) IP3-DAG and subsequent release of calcium, leading to synaptic plasticity; ii) PI3K/Akt and transcription of prosurvival genes and iii) MAPK/ERK and activation of differentiation promoting substrates [41]. With low affinity and also in immature form (as proneurotrophins) they interact with p75^NTR ^- a tumor necrosis factor receptor which, in turn, upon activation, leads to apoptosis in neuronal and non-neuronal cells [42]. Glial -Derived Neurotrophic Factor (GDNF) is a growth factor from the transforming growth factor β (TGFβ) superfamily, with documented neuroprotective effects in dopaminergic neurons cell cultures [43], *in vivo *studies on laboratory animals [44] and in animal models of PD [45,46]. It exerts its effects through Ret receptor tyrosine kinase and GDNF family receptor α1 (GFRα1) complex [47], although the role of Ret signaling is controversial [48,49]. Mesencephalic Astrocyte-Derived Neurotrophic Factor (MANF) and Conserved Dopamine Neurotrophic Factor (CDNF) are members of a novel, evolutionarily conserved neurotrophic factor family with specific protective properties on dopaminergic neurons, as shown in 6-hydroxydopamine (6-OHDA) animal models of PD [50]. Furthermore, they seem to act more effectively than GDNF and use a different protective mechanism [51].

#### Neurotrophins and growth factors in neurodegeneration

In both AD and PD human brains, levels of BDNF [52] and its mRNA [53] are low. Furthermore, BDNF serum levels correlate with AD severity [54]. Correlated alteration in TrkB expression in AD is also reported in cortical neurons, but not in glial cells, which, surprisingly, upregulate a truncated form of the receptor [55]. According to Tong et al., BDNF signaling pathway seems also to be negatively affected in AD, by Aβ 1-42 peptide interference with gene transcription. Treatment of rat cortical neurons cultures with sublethal doses of Aβ peptide, interfered with the CREB activation-induced transcription of the BDNF gene and suppressed BDNF-induced activation of selective signaling pathways such as Ras-MAPK/ERK and PI3-K/Akt [56].

The reports regarding NGF mRNA and protein levels in AD brain are contradictory [57-59]. NGF deficiency has been proposed as ethiopatogenic factor in sporadic AD, and the AD11 anti-NGF mice recreate the phenotype and the functional impairment of early AD stages [55]. Also, in early stages, a loss of TrkA has been reported [60], while Cuello et Bruno proposed the existence of a failure of the NGF maturation cascade in AD [61]. Aβ load recreates the same NGF "dismetabolism" in the hippocampus of laboratory rats, as proposed by Cuello et al. [62]. *In vitro *models showed Aβ peptide as a potent NGF -secretion stimulator in astrocytic rat cultures and, in turn, NGF was shown to increase neurotoxic potency of amyloid peptide in primary rat hippocampal cultures via p75 induction [63].

It is well documented that brains of PD patients express lower GDNF levels [64] and growth factor delivery in brain of PD animal models exerts neuroprotective effects and improves clinical outcome [65,66]. Furthermore, Sun et al. demonstrated in a rat model that GDNF is more efficient than BDNF in protecting striatal neurons from 6-hydroxydopamine (6-OHDA), compared to the control group or BDNF group. Moreover, simultaneous administration of both growth factors showed no benefit over GDNF treatment alone [67]. However, using vector-induced striatal neuron-restricted expression of both GDNF and BDNF genes, Cao et al. reported an improved protein expression as to either approach alone [68].

In human AD studies, there are controversial reports of GDNF protein levels. Straten et al. reported higher CSF concentration than age-matched controls along with decreased serum concentration [69], whereas Marksteiner's et al. results showed increased plasma levels in AD and mild cognitive impairment (MCI) patients [70]. However, in light of the serious side effects reported after intracerebroventricular infusion of GDNF in parkinsonian patients [71], attention was drown toward MANF and CDNF, which will hopefully make good candidates for novel therapies in PD.

### Neuroplasticity in neurodegeneration

Neuroplasticity is a comprehensive term that illustrates the brain's capacity to adapt, structurally and functionally, to environmental enhancement. According to Thickbroom and Mastaglia, the molecular mechanisms underlying neuroplasticity are both neuronal and non-neuronal and, furthermore, neuronal plasticity may be synaptic or non-synaptic [72]. Neuroplasticity is substrate for learning and memory formation, cognitive abilities progressively lost in AD and in late stages of PD.

Synaptic loss is one of the neurobiological hallmarks of AD, from the first stages of the disease [73]. The synaptic dysfunction is apparently due to soluble Aβ oligomers, as proven by studies on human AD brains [74] and AD animal models [75]. Soluble Aβ oligomers have a proven inhibitory effect on NMDA-R - dependent LTP [76], impairing even further the neuroplasticity, besides their roles in morphological and structural degeneration of the synapse [77].

Synapse alteration is initially compensated by "dynamic synaptic reorganization", emphasized by a paradoxical initial increase in synaptic markers [78]. The proof of network reorganization is sustained by studies on AD brains showing increased polysialylated neural cell adhesion molecule (PSA-NCAM) in dentate gyrus, as compared to controls [79]. Also investigating NCAM, Jørgensen et al hypothesize that AD brain uses neuroplasticity as a compensatory measure for neuronal loss [80]. Furthermore, inflammatory environment - a constant finding in AD brain - impairs neuronal plasticity by inhibiting both (NMDA-R) - induced and voltage-dependent calcium channel (VDCC)-induced LTP [81].

The other neuropathological hallmark of AD, tau hyperphosphorylation, correlates with low neuronal plasticity and synaptic disorganization, as proven by studies on hibernating animals [82]. Possibly a protective mechanism against neuronal apoptosis in unfavorable conditions, persistent hyperphosphorylation will eventually lead to formation of paired helical filaments and cell destruction.

PD animal models also show impaired neuroplasticity. Studies in mice overexpressing human α-synuclein report both short-term and long-term altered presynaptic plasticity in the corticostriatal pathway [83]. Transgenic mice bearing mutated α-synuclein - (A30P) α-synuclein - also showed impaired short-time synaptic plasticity [84] and the (6-OHDA) PD animal models develop defective synaptic plasticity induction [85]. Morphological studies of idiopathic PD brains and PD animal models reported that loss of dopaminergic input on medium spiny neurons of striatum resulted in lowerment of dendritic length, dendritic spine density, and total number of dendritic spines [86].

To conclude so far, there is evidence of impaired neural plasticity in both AD [87] and PD [86] brains, which occurs on various molecular levels, from growth factors signaling to synaptic malfunction, disorganization and cytoskeletal rearrangement. However, the brain possesses a latent recovery capacity and in early stages some compensatory mechanisms are triggered (see Table 1). Furthermore, the brain's capacity to compensate these structural and functional deficits is exploited by neurorestoration attempts in animal models and patients, as discussed below.

**Table 1:** Evidences of impaired neuroregeneration in AD and PD

Neurorestorative field		Evidence of impairment	Evidence of compensatory mechanism
Neurogenesis	AD	Decreased number of NPCs and altered division rates [21]	Increased neuroproliferation markers [16]

	PD	Reduced number of NPCs [28]	Increased number of PSA-NCAM + cells [29]

Neuroprotection	AD	Low BDNF mRNA and protein levels [37]	Upregulation of glial truncated TrkB [40]
		Controversed data on NGF levels [40,48,49]	Possibly upregulation of NGF with ageing and dementia [61,62,87]
			Aβ stimulates NGF astrocytic secrection [51]
			High GDNF levels in cerebrospinal fluid

	PD	Low BDNF mRNA and protein levels [37]	BDNF pretreatment protects dopaminergic neurons [34]
		Low GDNF protein levels [66]	

Neuroplasticity	AD	Synaptic loss [77]	"Dynamic synaptic reorganization" [82].
		LTP impairment by Abeta oligomers and inflammatory environment [85]	NCAM increase in dentate gyrus [83]

	PD	Loss of dendritic spines following loss of dopaminergic input [90]	
		Impaired synaptic plasticity in several models of PD [87-89]	

### Neurorestoration

At the base of initial neurorestoration attempts lies the idea of enhancing the endogenous neuroprotective effect of growth factors in the CNS. At first, genetically modified fibroblasts to produce either BDNF, or NGF have been transplanted in laboratory rats [88,89] and primates [90]. The experiments were successful in rescuing functional and cellular loss. The same type of experiment was conducted, in 2005, on human patients, diagnosed with AD [91]. The delivery system consisted of induced pluripotent stem cells (iPS), generated from the recipient's fibroblast population and genetically modified into secreting NGF. The authors reported significant progress at 22 months follow-up, quantified by cognitive scales and PET -Scan.

For PD patients, there are reports since the 1980's of fetal midbrain dopamine cells implants [92]. The clinical outcome was improved [93,94] and engraftment of transplanted cells was successful [95,96], although some authors questioned the utility of the procedure in older patients [97]. However, two double-blinded, randomized, controlled trials set back the initial positivism, showing cell transplantation to be less effective than deep brain stimulation [98], in preventing recurrent dyskinesia. It seems however, that reported improvement is due to replacement by graft cells of aged brain cells [99], rather than stimulation of the brain's own neurorestorative mechanism.

Other restorative models, tested *in vitro *or in animal models of AD and PD, use stem cells therapy: i) embryonic stem cells [100]; ii) embryonic stem cells-derived neurospheres [101]; iii) transdifferentiated stem cells (stem cells forced to differentiate outside their lineage by special growth media and specific stimuli) (e.g. hematopoietic stem cells), or iv) mesenchimal stem cells induced into secreting increased quantities of growth factors [102]. Apel et al. report neuroprotective effects of dental pulp cells co-cultured with hippocampal and mesencephalic rat neurons, in *in vitro *AD and PD models [103]. Murell et al used human olfactory mucosa-derived neuronal progenitors to obtain dopaminergic neurons and transplant them in a rat PD model brain. The outcome was favorable and no difference was noted between transplants received form healthy donors or from Parkinson patients [104].

## Summary

As expected, most reports incline towards progressive impairment of neuroregeneration resources in AD and PD brains, as proven on human post-mortem analysis, animal models and *in vitro *studies. However, due to increased amount of evidence that proper stimulation or supply of growth factors restores some of the cognitive loss and ameliorates behavioral skills, we hypothesize that the brain possess, at least in early and medium stages of disease, a "neuroregenerative reserve", that may be and begins to be, targeted as a therapeutical perspective.

## List of abbreviations

AD: Alzheimer's Disease; PD: Parkinson's Disease; NPCs: neural precursor cells; PSA-NCAM: Polysialylated Neural Cell Adhesion Molecule; BDNF: Brain Derived Nerve Factor; TrkB: tropomyosin-related kinase receptor B; NGF: Nerve Growth Factor; GDNF: Glial Derived Nerve Factor

## Competing interests

The authors declare that they have no competing interests.

## Authors' contributions

All authors contributed equally to elaboration of the manuscript, read and approved the final manuscript.

## Pre-publication history

The pre-publication history for this paper can be accessed here:

http://www.biomedcentral.com/1471-2377/11/75/prepub
